# Supplementary material for: Modeling glioblastoma heterogeneity as a dynamic network of cell states
Source: Mol Syst Biol. 2021 Sep 16;17(9):e10105. doi: 10.15252/msb.202010105 (PMC8444284; doi:10.15252/msb.202010105)
Supplement: Supplementary file 6 — Source Data for Figure 5 [file MSB-17-e10105-s004.zip › Figure5A_sourcedata/GSEA_3071/hallmarks_stateIV.GseaPreranked.1623742551702/HALLMARK_HEME_METABOLISM.html]

Details for gene set HALLMARK\_HEME\_METABOLISM[GSEA]

|  || Dataset | state53071 |
| Phenotype | NoPhenotypeAvailable |
| Upregulated in class | na\_pos |
| GeneSet | HALLMARK\_HEME\_METABOLISM |
| Enrichment Score (ES) | 0.17558776 |
| Normalized Enrichment Score (NES) | 0.90147024 |
| Nominal p-value | 0.6169725 |
| FDR q-value | 0.88577884 |
| FWER p-Value | 1.0 |
Table: GSEA Results Summary

  

Fig 1: Enrichment plot: HALLMARK\_HEME\_METABOLISM      
 Profile of the Running ES Score & Positions of GeneSet Members on the Rank Ordered List

  

| PROBE | GENE SYMBOL | GENE\_TITLE | RANK IN GENE LIST | RANK METRIC SCORE | RUNNING ES | CORE ENRICHMENT || 1 | PRDX2 |  |  | 143 | 1.387 | 0.0042 | Yes |
| 2 | IGSF3 |  |  | 173 | 1.309 | 0.0284 | Yes |
| 3 | BTG2 |  |  | 200 | 1.260 | 0.0521 | Yes |
| 4 | LMO2 |  |  | 272 | 1.099 | 0.0635 | Yes |
| 5 | FOXO3 |  |  | 291 | 1.054 | 0.0841 | Yes |
| 6 | DAAM1 |  |  | 444 | 0.860 | 0.0745 | Yes |
| 7 | GYPC |  |  | 474 | 0.833 | 0.0879 | Yes |
| 8 | TFDP2 |  |  | 527 | 0.789 | 0.0959 | Yes |
| 9 | HAGH |  |  | 547 | 0.766 | 0.1097 | Yes |
| 10 | ADD2 |  |  | 572 | 0.747 | 0.1221 | Yes |
| 11 | RNF19A |  |  | 649 | 0.692 | 0.1233 | Yes |
| 12 | MBOAT2 |  |  | 664 | 0.679 | 0.1361 | Yes |
| 13 | RBM5 |  |  | 697 | 0.662 | 0.1450 | Yes |
| 14 | TNRC6B |  |  | 698 | 0.662 | 0.1601 | Yes |
| 15 | CLCN3 |  |  | 759 | 0.624 | 0.1628 | Yes |
| 16 | AGPAT4 |  |  | 767 | 0.622 | 0.1756 | Yes |
| 17 | RBM38 |  |  | 902 | 0.560 | 0.1627 | No |
| 18 | MXI1 |  |  | 944 | 0.546 | 0.1672 | No |
| 19 | NUDT4 |  |  | 991 | 0.525 | 0.1704 | No |
| 20 | ARHGEF12 |  |  | 1172 | 0.473 | 0.1467 | No |
| 21 | DCUN1D1 |  |  | 1202 | 0.464 | 0.1517 | No |
| 22 | BSG |  |  | 1225 | 0.458 | 0.1579 | No |
| 23 | ATP6V0A1 |  |  | 1320 | 0.436 | 0.1498 | No |
| 24 | SLC6A8 |  |  | 1365 | 0.424 | 0.1510 | No |
| 25 | ADD1 |  |  | 1432 | 0.409 | 0.1477 | No |
| 26 | KLF3 |  |  | 1437 | 0.406 | 0.1562 | No |
| 27 | NARF |  |  | 1692 | 0.351 | 0.1155 | No |
| 28 | TFRC |  |  | 1750 | 0.339 | 0.1123 | No |
| 29 | TMEM9B |  |  | 1771 | 0.336 | 0.1162 | No |
| 30 | UROS |  |  | 2105 | 0.283 | 0.0588 | No |
| 31 | VEZF1 |  |  | 2161 | 0.274 | 0.0545 | No |
| 32 | GDE1 |  |  | 2269 | 0.260 | 0.0399 | No |
| 33 | TCEA1 |  |  | 2385 | -0.256 | 0.0237 | No |
| 34 | SLC10A3 |  |  | 2429 | -0.261 | 0.0214 | No |
| 35 | SLC22A4 |  |  | 2536 | -0.273 | 0.0074 | No |
| 36 | BACH1 |  |  | 2581 | -0.280 | 0.0053 | No |
| 37 | UCP2 |  |  | 2602 | -0.283 | 0.0079 | No |
| 38 | PSMD9 |  |  | 2647 | -0.289 | 0.0061 | No |
| 39 | MFHAS1 |  |  | 3067 | -0.341 | -0.0664 | No |
| 40 | SMOX |  |  | 3075 | -0.342 | -0.0600 | No |
| 41 | BMP2K |  |  | 3095 | -0.345 | -0.0558 | No |
| 42 | LRP10 |  |  | 3175 | -0.356 | -0.0628 | No |
| 43 | HDGF |  |  | 3181 | -0.357 | -0.0556 | No |
| 44 | GLRX5 |  |  | 3225 | -0.364 | -0.0556 | No |
| 45 | ENDOD1 |  |  | 3247 | -0.368 | -0.0512 | No |
| 46 | UROD |  |  | 3315 | -0.377 | -0.0555 | No |
| 47 | RIOK3 |  |  | 3484 | -0.406 | -0.0784 | No |
| 48 | CDR2 |  |  | 3486 | -0.407 | -0.0693 | No |
| 49 | TSPAN5 |  |  | 3515 | -0.412 | -0.0653 | No |
| 50 | PICALM |  |  | 3616 | -0.431 | -0.0747 | No |
| 51 | MAP2K3 |  |  | 3674 | -0.439 | -0.0756 | No |
| 52 | CCND3 |  |  | 3901 | -0.487 | -0.1078 | No |
| 53 | MGST3 |  |  | 3917 | -0.491 | -0.0995 | No |
| 54 | CDC27 |  |  | 3918 | -0.491 | -0.0883 | No |
| 55 | HEBP1 |  |  | 4012 | -0.516 | -0.0944 | No |
| 56 | BPGM |  |  | 4213 | -0.570 | -0.1197 | No |
| 57 | BLVRA |  |  | 4228 | -0.574 | -0.1093 | No |
| 58 | BCAM |  |  | 4346 | -0.614 | -0.1178 | No |
| 59 | EPB41 |  |  | 4449 | -0.654 | -0.1224 | No |
| 60 | BLVRB |  |  | 4533 | -0.698 | -0.1224 | No |
| 61 | NEK7 |  |  | 4604 | -0.734 | -0.1191 | No |
| 62 | NR3C1 |  |  | 4699 | -0.796 | -0.1190 | No |
| 63 | ELL2 |  |  | 4935 | -1.000 | -0.1412 | No |
| 64 | CTSB |  |  | 4948 | -1.012 | -0.1205 | No |
| 65 | SLC2A1 |  |  | 4949 | -1.018 | -0.0973 | No |
| 66 | OPTN |  |  | 4995 | -1.083 | -0.0813 | No |
| 67 | GCLM |  |  | 5077 | -1.235 | -0.0687 | No |
| 68 | CAST |  |  | 5093 | -1.274 | -0.0426 | No |
| 69 | P4HA2 |  |  | 5127 | -1.346 | -0.0183 | No |
| 70 | SLC7A11 |  |  | 5257 | -2.165 | 0.0063 | No |
Table: GSEA details [plain text format]

  

Fig 2: HALLMARK\_HEME\_METABOLISM: Random ES distribution      
 Gene set null distribution of ES for **HALLMARK\_HEME\_METABOLISM**
